# Radiogenomics of glioblastoma: a window into its imaging and molecular variability

**DOI:** 10.1186/1470-7330-15-S1-P14

**Published:** 2015-10-02

**Authors:** A Mahajan, A V Moiyadi, R Jalali, E Sridhar

**Affiliations:** 1 Tata Memorial Centre, Mumbai, India

## Learning objectives

Gliomas, the most frequent tumours occurring in the CNS, are defined and graded on the basis of histological features to predict prognosis and management. Owing to biological heterogeneity, the histological diagnosis and expected clinical outcome do not match in a significant number of patients. The combination of imaging and gene expression, referred to as “radiogenomics,” has the potential to give insight into tumour biology that would be difficult to acquire from either technique alone.

• Throwing light upon individual imaging feature and biological/ molecular alterations, analyses that may detect subsets of morphologically identical tumours with different clinical behavior (diagnostic markers).

## Content organisation

• Classification and grading of Gliomas as per Haarlem consensus.

• Imaging features of glioblastomas may correlate with molecular mutations.

• Identification of imaging parameters that has the potential as a non-invasive biomarker for common molecular mutations associated with gliomas and their associated clinical impact.

• Review of current radiogenomics literature.

## Conclusion

• Given the noninvasive nature of medical imaging and its wide use in clinical practice this approach facilitates the association of complex molecular signatures with readily identifiable imaging characteristics.

• As we move toward a more individualised approach to therapy for glioblastoma on the basis of its specific genetic and biochemical features, radiologists may contribute to the future development of targeted agents for treatment of glioblastoma.

